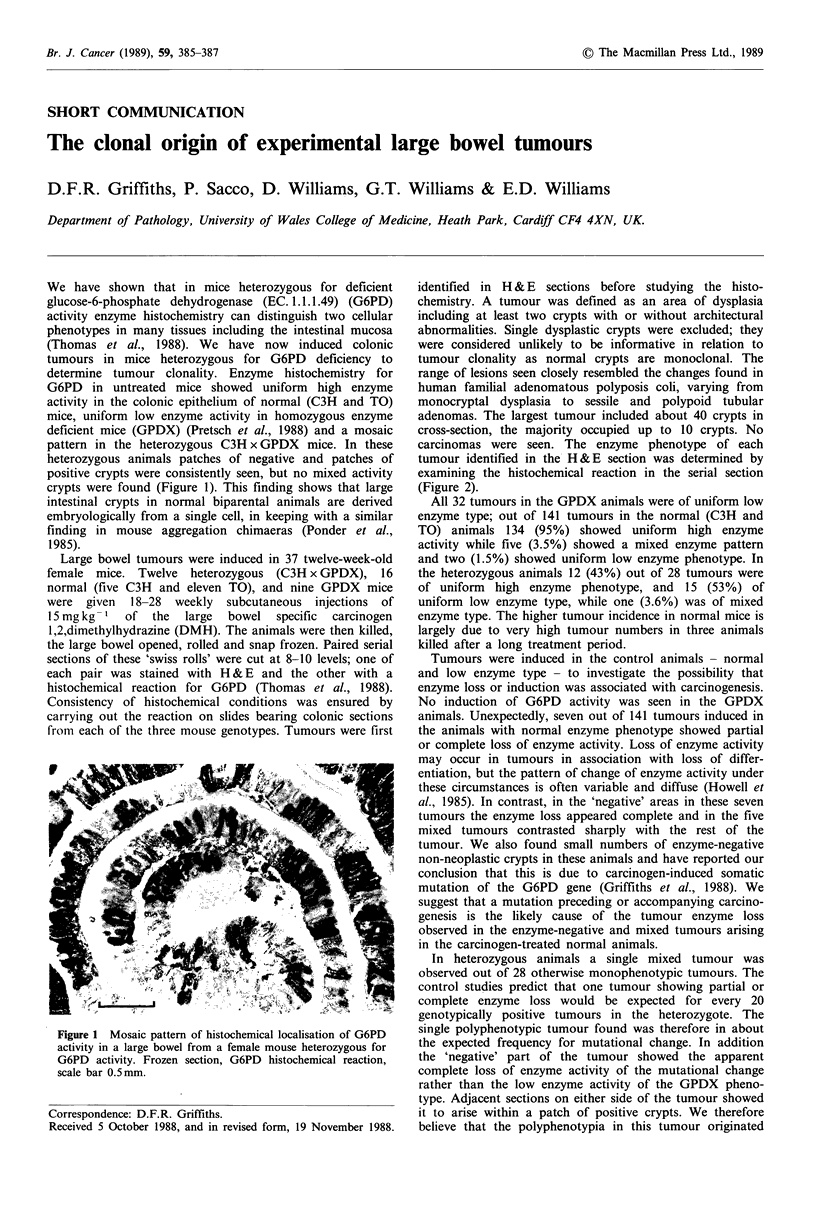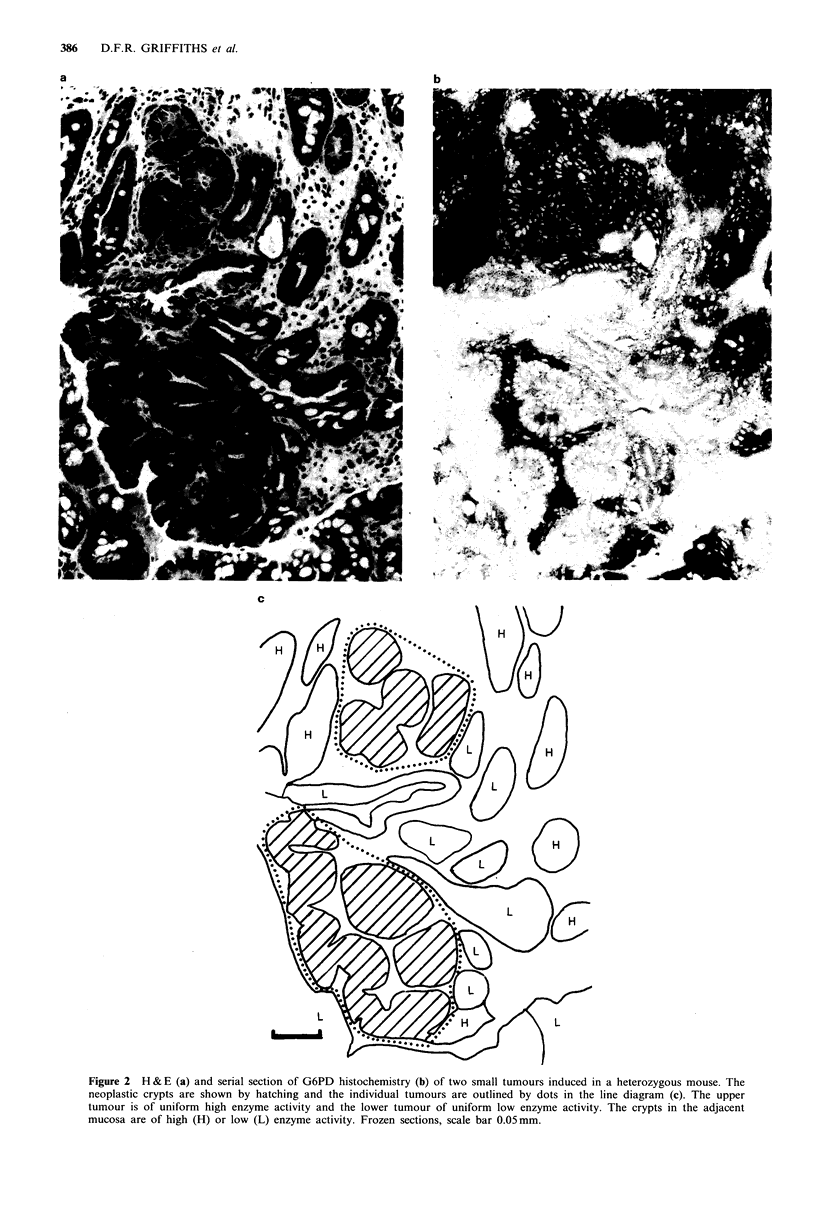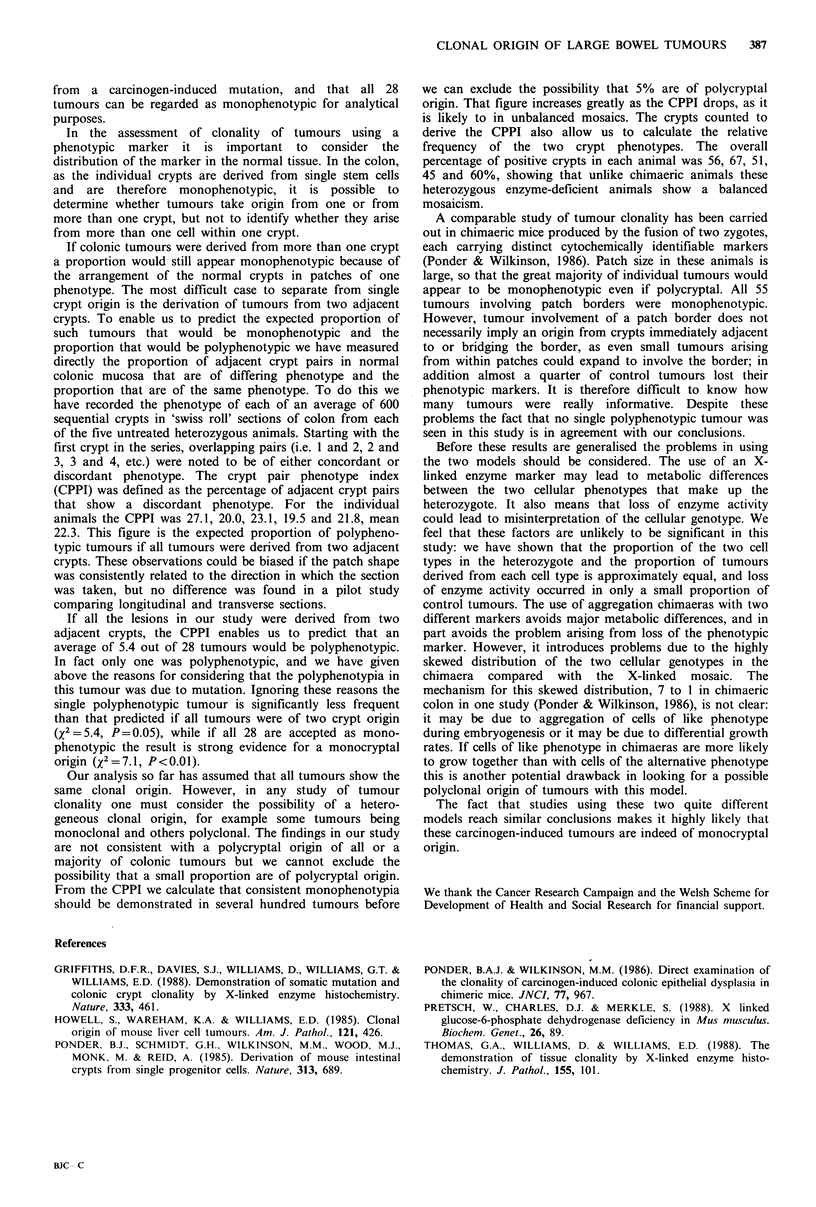# The clonal origin of experimental large bowel tumours.

**DOI:** 10.1038/bjc.1989.77

**Published:** 1989-03

**Authors:** D. F. Griffiths, P. Sacco, D. Williams, G. T. Williams, E. D. Williams

**Affiliations:** Department of Pathology, University of Wales College of Medicine, Cardiff, UK.

## Abstract

**Images:**


Br. J. Cancer (1989), 59, 385–387 © The Macmillan Press Ltd., 1989

SHORT COMMUNICATION

### The clonal origin of experimental large bowel tumours

D.F.R. Griffiths, P. Sacco, D. Williams, G.T. Williams & E.D. Williams

*Department of Pathology, University of Wales College of Medicine, Heath Park, Cardiff CF4 4XN, UK.*

We have shown that in mice heterozygous for deficient glucose-6-phosphate dehydrogenase (EC. 1.1.1.49) (G6PD) activity enzyme histochemistry can distinguish two cellular phenotypes in many tissues including the intestinal mucosa (Thomas *et al.*, 1988). We have now induced colonic tumours in mice heterozygous for G6PD deficiency to determine tumour clonality. Enzyme histochemistry for G6PD in untreated mice showed uniform high enzyme activity in the colonic epithelium of normal (C3H and TO) mice, uniform low enzyme activity in homozygous enzyme deficient mice (GPDX) (Pretsch *et al.*, 1988) and a mosaic pattern in the heterozygous C3H x GPDX mice. In these heterozygous animals patches of negative and patches of positive crypts were consistently seen, but no mixed activity crypts were found (Figure 1). This finding shows that large intestinal crypts in normal biparental animals are derived embryologically from a single cell, in keeping with a similar finding in mouse aggregation chimaeras (Ponder *et al.*, 1985).

Large bowel tumours were induced in 37 twelve-week-old female mice. Twelve heterozygous (C3H x GPDX), 16 normal (five C3H and eleven TO), and nine GPDX mice were given 18–28 weekly subcutaneous injections of $15\,mg\,kg^{-1}$ of the large bowel specific carcinogen 1,2,dimethylhydrazine (DMH). The animals were then killed, the large bowel opened, rolled and snap frozen. Paired serial sections of these 'swiss rolls' were cut at 8–10 levels; one of each pair was stained with H&E and the other with a histochemical reaction for G6PD (Thomas *et al.*, 1988). Consistency of histochemical conditions was ensured by carrying out the reaction on slides bearing colonic sections from each of the three mouse genotypes. Tumours were first identified in H&E sections before studying the histochemistry. A tumour was defined as an area of dysplasia including at least two crypts with or without architectural abnormalities. Single dysplastic crypts were excluded; they were considered unlikely to be informative in relation to tumour clonality as normal crypts are monoclonal. The range of lesions seen closely resembled the changes found in human familial adenomatous polyposis coli, varying from monocryptal dysplasia to sessile and polypoid tubular adenomas. The largest tumour included about 40 crypts in cross-section, the majority occupied up to 10 crypts. No carcinomas were seen. The enzyme phenotype of each tumour identified in the H&E section was determined by examining the histochemical reaction in the serial section (Figure 2).

**Figure 1** Mosaic pattern of histochemical localisation of G6PD activity in a large bowel from a female mouse heterozygous for G6PD activity. Frozen section, G6PD histochemical reaction, scale bar 0.5 mm.

All 32 tumours in the GPDX animals were of uniform low enzyme type; out of 141 tumours in the normal (C3H and TO) animals 134 (95%) showed uniform high enzyme activity while five (3.5%) showed a mixed enzyme pattern and two (1.5%) showed uniform low enzyme phenotype. In the heterozygous animals 12 (43%) out of 28 tumours were of uniform high enzyme phenotype, and 15 (53%) of uniform low enzyme type, while one (3.6%) was of mixed enzyme type. The higher tumour incidence in normal mice is largely due to very high tumour numbers in three animals killed after a long treatment period.

Tumours were induced in the control animals – normal and low enzyme type – to investigate the possibility that enzyme loss or induction was associated with carcinogenesis. No induction of G6PD activity was seen in the GPDX animals. Unexpectedly, seven out of 141 tumours induced in the animals with normal enzyme phenotype showed partial or complete loss of enzyme activity. Loss of enzyme activity may occur in tumours in association with loss of differentiation, but the pattern of change of enzyme activity under these circumstances is often variable and diffuse (Howell *et al.*, 1985). In contrast, in the 'negative' areas in these seven tumours the enzyme loss appeared complete and in the five mixed tumours contrasted sharply with the rest of the tumour. We also found small numbers of enzyme-negative non-neoplastic crypts in these animals and have reported our conclusion that this is due to carcinogen-induced somatic mutation of the G6PD gene (Griffiths *et al.*, 1988). We suggest that a mutation preceding or accompanying carcinogenesis is the likely cause of the tumour enzyme loss observed in the enzyme-negative and mixed tumours arising in the carcinogen-treated normal animals.

In heterozygous animals a single mixed tumour was observed out of 28 otherwise monophenotypic tumours. The control studies predict that one tumour showing partial or complete enzyme loss would be expected for every 20 genotypically positive tumours in the heterozygote. The single polyphenotypic tumour found was therefore in about the expected frequency for mutational change. In addition the 'negative' part of the tumour showed the apparent complete loss of enzyme activity of the mutational change rather than the low enzyme activity of the GPDX phenotype. Adjacent sections on either side of the tumour showed it to arise within a patch of positive crypts. We therefore believe that the polyphenotypia in this tumour originated

Correspondence: D.F.R. Griffiths.
Received 5 October 1988, and in revised form, 19 November 1988.

**Figure 2** H & E (**a**) and serial section of G6PD histochemistry (**b**) of two small tumours induced in a heterozygous mouse. The neoplastic crypts are shown by hatching and the individual tumours are outlined by dots in the line diagram (**c**). The upper tumour is of uniform high enzyme activity and the lower tumour of uniform low enzyme activity. The crypts in the adjacent mucosa are of high (H) or low (L) enzyme activity. Frozen sections, scale bar 0.05 mm.

from a carcinogen-induced mutation, and that all 28 tumours can be regarded as monophenotypic for analytical purposes.

In the assessment of clonality of tumours using a phenotypic marker it is important to consider the distribution of the marker in the normal tissue. In the colon, as the individual crypts are derived from single stem cells and are therefore monophenotypic, it is possible to determine whether tumours take origin from one or from more than one crypt, but not to identify whether they arise from more than one cell within one crypt.

If colonic tumours were derived from more than one crypt a proportion would still appear monophenotypic because of the arrangement of the normal crypts in patches of one phenotype. The most difficult case to separate from single crypt origin is the derivation of tumours from two adjacent crypts. To enable us to predict the expected proportion of such tumours that would be monophenotypic and the proportion that would be polyphenotypic we have measured directly the proportion of adjacent crypt pairs in normal colonic mucosa that are of differing phenotype and the proportion that are of the same phenotype. To do this we have recorded the phenotype of each of an average of 600 sequential crypts in 'swiss roll' sections of colon from each of the five untreated heterozygous animals. Starting with the first crypt in the series, overlapping pairs (i.e. 1 and 2, 2 and 3, 3 and 4, etc.) were noted to be of either concordant or discordant phenotype. The crypt pair phenotype index (CPPI) was defined as the percentage of adjacent crypt pairs that show a discordant phenotype. For the individual animals the CPPI was 27.1, 20.0, 23.1, 19.5 and 21.8, mean 22.3. This figure is the expected proportion of polyphenotypic tumours if all tumours were derived from two adjacent crypts. These observations could be biased if the patch shape was consistently related to the direction in which the section was taken, but no difference was found in a pilot study comparing longitudinal and transverse sections.

If all the lesions in our study were derived from two adjacent crypts, the CPPI enables us to predict that an average of 5.4 out of 28 tumours would be polyphenotypic. In fact only one was polyphenotypic, and we have given above the reasons for considering that the polyphenotypia in this tumour was due to mutation. Ignoring these reasons the single polyphenotypic tumour is significantly less frequent than that predicted if all tumours were of two crypt origin ($\chi^2=5.4$, $P=0.05$), while if all 28 are accepted as monophenotypic the result is strong evidence for a monocryptal origin ($\chi^2=7.1$, $P<0.01$).

Our analysis so far has assumed that all tumours show the same clonal origin. However, in any study of tumour clonality one must consider the possibility of a heterogeneous clonal origin, for example some tumours being monoclonal and others polyclonal. The findings in our study are not consistent with a polycryptal origin of all or a majority of colonic tumours but we cannot exclude the possibility that a small proportion are of polycryptal origin. From the CPPI we calculate that consistent monophenotypia should be demonstrated in several hundred tumours before we can exclude the possibility that 5% are of polycryptal origin. That figure increases greatly as the CPPI drops, as it is likely to in unbalanced mosaics. The crypts counted to derive the CPPI also allow us to calculate the relative frequency of the two crypt phenotypes. The overall percentage of positive crypts in each animal was 56, 67, 51, 45 and 60%, showing that unlike chimaeric animals these heterozygous enzyme-deficient animals show a balanced mosaicism.

A comparable study of tumour clonality has been carried out in chimaeric mice produced by the fusion of two zygotes, each carrying distinct cytochemically identifiable markers (Ponder & Wilkinson, 1986). Patch size in these animals is large, so that the great majority of individual tumours would appear to be monophenotypic even if polycryptal. All 55 tumours involving patch borders were monophenotypic. However, tumour involvement of a patch border does not necessarily imply an origin from crypts immediately adjacent to or bridging the border, as even small tumours arising from within patches could expand to involve the border; in addition almost a quarter of control tumours lost their phenotypic markers. It is therefore difficult to know how many tumours were really informative. Despite these problems the fact that no single polyphenotypic tumour was seen in this study is in agreement with our conclusions.

Before these results are generalised the problems in using the two models should be considered. The use of an X-linked enzyme marker may lead to metabolic differences between the two cellular phenotypes that make up the heterozygote. It also means that loss of enzyme activity could lead to misinterpretation of the cellular genotype. We feel that these factors are unlikely to be significant in this study: we have shown that the proportion of the two cell types in the heterozygote and the proportion of tumours derived from each cell type is approximately equal, and loss of enzyme activity occurred in only a small proportion of control tumours. The use of aggregation chimaeras with two different markers avoids major metabolic differences, and in part avoids the problem arising from loss of the phenotypic marker. However, it introduces problems due to the highly skewed distribution of the two cellular genotypes in the chimaera compared with the X-linked mosaic. The mechanism for this skewed distribution, 7 to 1 in chimaeric colon in one study (Ponder & Wilkinson, 1986), is not clear: it may be due to aggregation of cells of like phenotype during embryogenesis or it may be due to differential growth rates. If cells of like phenotype in chimaeras are more likely to grow together than with cells of the alternative phenotype this is another potential drawback in looking for a possible polyclonal origin of tumours with this model.

The fact that studies using these two quite different models reach similar conclusions makes it highly likely that these carcinogen-induced tumours are indeed of monocryptal origin.

We thank the Cancer Research Campaign and the Welsh Scheme for Development of Health and Social Research for financial support.

### References

GRIFFITHS, D.F.R., DAVIES, S.J., WILLIAMS, D., WILLIAMS, G.T. & WILLIAMS, E.D. (1988). Demonstration of somatic mutation and colonic crypt clonality by X-linked enzyme histochemistry. *Nature*, **333**, 461.

HOWELL, S., WAREHAM, K.A. & WILLIAMS, E.D. (1985). Clonal origin of mouse liver cell tumours. *Am. J. Pathol.*, **121**, 426.

PONDER, B.J., SCHMIDT, G.H., WILKINSON, M.M., WOOD, M.J., MONK, M. & REID, A. (1985). Derivation of mouse intestinal crypts from single progenitor cells. *Nature*, **313**, 689.

PONDER, B.A.J. & WILKINSON, M.M. (1986). Direct examination of the clonality of carcinogen-induced colonic epithelial dysplasia in chimeric mice. *JNCI*, **77**, 967.

PRETSCH, W., CHARLES, D.J. & MERKLE, S. (1988). X linked glucose-6-phosphate dehydrogenase deficiency in *Mus musculus*. *Biochem. Genet.*, **26**, 89.

THOMAS, G.A., WILLIAMS, D. & WILLIAMS, E.D. (1988). The demonstration of tissue clonality by X-linked enzyme histochemistry. *J. Pathol.*, **155**, 101.